# Symptom Burden and Patient-Reported Outcomes in Kidney Transplant Recipients: Results From the TransplantLines Biobank and Cohort Studies

**DOI:** 10.1016/j.xkme.2025.101168

**Published:** 2025-10-31

**Authors:** Niels L. Riemersma, Tim J. Knobbe, Daan Kremer, Svea Nolte, Ute Bültmann, Coby Annema, Naser B.N. Shehab, Michele F. Eisenga, Stefan P. Berger, Stephan J.L. Bakker, TransplantLines Investigators, C. Annema, S.J.L. Bakker, S.P. Berger, H. Blokzijl, F.A.J.A. Bodewes, M.T. de Boer, Damman, M.H. de Borst, A. Diepstra, G. Dijkstra, R.M. Douwes, C.S.E. Doorenbos, M.F. Eisenga, M.E. Erasmus, C.T. Gan, A.W.Gomes Neto, A.M. Posthumus, E. Hak, B.G. Hepkema, J. Jonker, F. Klont, T.J. Knobbe, D. Kremer, H.G.D. Leuvenink, W.S. Lexmond, V.E. de Meijer, G.J. Nieuwenhuis-Moeke, H.G.M. Niesters, L.J. van Pelt, R.A. Pol, A.V. Ranchor, J.S.F. Sanders, M.J. Siebelink, R.J.H.J.A. Slart, J.C. Swarte, D.J. Touw, M.C. van den Heuvel, C. van Leer-Buter, M. van Londen, Charlotte A. te Velde-Keyzer, E.A.M. Verschuuren, M.J. Vos, R.K. Weersma

**Affiliations:** 1 University of Groningen, University Medical Center Groningen, Department of Internal Medicine, Division of Nephrology, Groningen, The Netherlands; 2 University of Groningen, University Medical Center Groningen, Community and Occupational Medicine, Groningen, The Netherlands; 3 University of Groningen, University Medical Center Groningen, Department of Health Sciences, Section of Nursing Science, Groningen, The Netherlands; 4 University Medical Center Groningen, UMC Groningen Transplant Center, Groningen, The Netherlands

**Keywords:** Health-related quality of life, kidney transplant recipients, medication nonadherence, patient-reported outcomes symptom burden, symptoms of anxiety, symptoms of depression

## Abstract

**Rationale & Objective:**

A multitude of symptoms may contribute to low health-related quality of life (HRQoL) in kidney transplant recipients (KTR). We aimed to identify the most occurring and distressing symptoms, to explore potential determinants of symptom burden, and to examine associations with patient-reported outcomes in KTR.

**Study Design:**

A cross-sectional retrospective patient-reported outcome measures study.

**Setting & Participants:**

Stable KTR ≥1 year after transplantation participating in the TransplantLines Biobank and Cohort Studies.

**Predictors:**

Clinical variables, including sex, age, and time after transplantation.

**Outcomes:**

Symptom occurrence/distress/burden, medication adherence, symptoms of depression/anxiety, societal participation, and HRQoL.

**Analytical Approach:**

Symptoms were evaluated using ridit analyses. A burden score was calculated to explore determinants of symptom burden and its associations with other patient-reported outcomes.

**Results:**

We included 936 KTR (38.8% female; mean ± SD age, 55.6 ± 13.0 years) at a median [IQR] of 2.0 [1.0-9.0] years after transplantation. Based on ridit scores, most occurring symptoms were tiredness [0.724], bruises [0.718], and lack of energy [0.688]; most distressful symptoms were menstrual problems [0.679], impotence [0.654], and joint pain [0.611]. Worse nutritional status (*P* < 0.001), being female (*P* < 0.001), cyclosporine use (*P* = 0.005), and proton pomp inhibitor use (*P* < 0.001) were associated with higher symptom burden. Higher symptom burden was associated with medication nonadherence, symptoms of depression and anxiety, lower societal participation, and lower physical and mental HRQoL (st.β = –0.53, 95% CI –0.59 to –0.47, *P* <0.001 and st.β=-0.53, 95% CI –0.60 to –0.46, *P* < 0.001, respectively).

**Limitations:**

No causality can be established because of the cross-sectional design.

**Conclusions:**

The most occurring symptoms were tiredness, bruises, and lack of energy, and the most distressing symptoms were menstrual problems, impotence, and joint pain. The strongest determinants of symptom burden were female sex, malnutrition, cyclosporine use, and proton pump inhibitor use. The associations of symptom burden with patient-related outcomes underline the importance of addressing symptom status after transplant.

## Plain-language Summary

Symptom burden of immunosuppressive medication may cause lower health-related quality of life (HRQoL) in kidney transplant recipients (KTR). Therefore, we aimed to assess (1) symptom occurrence and distress, (2) determinants of symptom burden, and (3) associations of symptom burden with HRQoL and other patient-reported outcomes. In 936 KTR, the most occurring symptoms were tiredness, bruises, and lack of energy, and the most distressing symptoms were menstrual problems, impotence, and joint pain. Being female, having malnutrition, using cyclosporine, and using proton pump inhibitors were determinants of high symptom burden. Furthermore, a higher symptom burden was associated with more medication nonadherence, more symptoms of anxiety/depression, lower societal participation, and lower physical and mental HRQoL. These findings may help improve patient-centered health care.

Kidney transplantation is the preferred treatment for kidney failure. It is superior to hemodialysis in terms of cost-effectiveness, life expectancy, and health-related quality of life (HRQoL).^1^, ^2^, ^3^, ^4^ However, HRQoL of kidney transplant recipients (KTR) remains lower compared with the general population, which may be attributable to the multitude of symptoms KTR experience.^5^, ^6^, ^7^, ^8^

Symptoms, such as tiredness, bruises, or tremor, may arise from underlying pathologies or as side effects from immunosuppressive medication. Little is known about which symptoms KTR experience as most occurring, most distressing, or both, and high occurrence does not always mean high distress. Additionally, symptoms with little direct effect on morbidity or mortality (eg, impotence) are prone to be neglected during consultations, but can be extremely distressing for patients.^9^ In addition, different patient groups, for example stratified by sex, age, or time after transplantation, may experience symptoms differently.^8^ These studies can be strengthened by adding ridit analyses. Mapping occurrence and distress of symptoms may help to identify which symptoms may be particularly important for patient well-being and may aid in improving personalized health care.

Furthermore, it is crucial to look at potential determinants of symptom burden. Women generally experience a higher symptom burden, and age and time since transplantation may also influence which symptoms are perceived as most burdensome.^7^^,^^8^^,^^10^^,^^11^ However, large studies with extensive analyses of potential determinants are lacking. In this study, we combine symptom occurrence with symptom distress to create a burden score, allowing for an extensive exploration of these potential determinants. The identification of these determinants of symptom burden is important, because a higher symptom occurrence and distress have been associated with medication nonadherence, depression, and lower HRQoL.^10^^,^^12^, ^13^, ^14^, ^15^ Medication nonadherence, in turn, may lead to complications, hospital admission, and even graft failure.^14^^,^^16^, ^17^, ^18^, ^19^ Other, yet unexamined, hypotheses are that symptom burden may be associated with anxiety or societal participation.

Therefore, we aimed to identify the most occurring and distressing symptoms in a large cohort of KTR and to explore potential differences in symptom experience between groups, stratified by sex, age, and time after transplantation. Secondly, we aimed to explore potential demographic, clinical and pharmacotherapeutic determinants of symptom burden. Lastly, we aimed to examine associations of symptom burden with medication nonadherence, symptoms of depression/anxiety, societal participation, and HRQoL.

## Materials and Methods

### Design and Study Population

We conducted a cross-sectional study with data from the TransplantLines Biobank and Cohort Study (ClinicalTrials.gov Identifier: NCT03272841).^20^ Since June 2015, all solid organ transplant recipients and donors aged ≥18 years old in the University Medical Center Groningen (Groningen, The Netherlands) were invited to participate in this study. The cohort has a participation rate of 81%. All participants signed written informed consent. The study was conducted in accordance with the guidelines laid down in the Declarations of Helsinki and Istanbul, and the study protocol was approved by the Institutional Review Board (METc 2014/077). Data extraction was performed in February 2022.

In the current study, we included outpatient KTR with a functioning graft approximately ≥1 year posttransplantation and with available data from the revised Modified Transplant Symptom Occurrence and Symptom Distress Scale (MTSOSD-59R) questionnaire. Patients were excluded from analyses if they completed <95% of the MTSOSD-59R or did not use immunosuppressants.

### Immunosuppressive Regimen

Participants were generally treated with standard immunosuppressive regimens, consisting of basiliximab-induced immunosuppression followed by triple drug maintenance therapy generally consisting of tacrolimus (target trough level: 4-6 μg/L), mycophenolate mofetil, and prednisolone. Cyclosporin (target trough level: 75-125 μg/L), azathioprine, and mTOR inhibitors were used less often. Beyond 1 year after transplantation, patients generally visit the transplantation outpatient clinic at least once yearly, with quarterly visits to the nephrologists in local hospitals.

### Symptom Burden

Symptoms were measured using the MTSOSD-59R questionnaire, which evaluates occurrence and distress of symptoms over the 4 weeks before the outpatient visit on a 5-point Likert scale (0 = never present/not at all distressing; 4 = always present/extremely distressing).^21^ Two questions are sex specific (impotence for men and menstrual problems for women), resulting in 59 questions per participant. An overview of the symptoms is provided in Table S1.

Symptom occurrence and symptom distress were assessed using ridit (relative to an identified distribution integral transformation) analyses. This nonparametric method is used to compare and rank ordinal data from multiple groups and has been described previously.^22^ A ridit score, ranging from 0 to 1, was calculated for the occurrence and distress of each symptom. The reference group equals a ridit score of 0.5. Thus, a ridit score of 0.750 on symptom occurrence of nausea means that if you pick a symptom at random from the reference group, there is a 75% chance it occurs less often than nausea. To simplify, a ridit score >0.5 indicates a higher occurrence or distress of the symptom, compared with the reference group, whereas a ridit score <0.5 indicates a lower occurrence or distress of the symptom, compared with the reference group. The reference groups were occurrence/distress of all symptoms of the total population and of each subgroup stratified by sex, age, and time after transplantation.

Symptom burden was calculated using the following formula: $\sum_{n=1\;}^{59}a_{n}*\left(b_{n}+1\right)$. This formula captures both symptoms occurrence and distress, and adding one point to the distress score ensures that the mere occurrence of symptoms also contributes to overall burden. This approach facilitates analyses of potential determinants of symptom burden and its associations with other patient-related outcomes. The symptom burden score ranges from 0 to 1,180 and was calculated for each patient. For example, when a patient reports to have headaches almost always (occurrence = 3 points) which are extremely distressing (distress = 4 points), the burden score of that symptom will be 3 × (4 + 1) = 15 points. Each symptom can have a maximum symptom burden score of 4 × (4 + 1) = 20. The total burden score is calculated by summing all individual symptoms.

### Outcomes and Additional Data Collection

A detailed description of the outcome variables and additional data collection can be found in Table S2. In short, medication nonadherence was assessed with the Basel Assessment of Adherence to Immunosuppressive Medication Scale (BAASIS), symptoms of depression were assessed with the Patient Health Questionnaire (PHQ-9), symptoms of anxiety were assessed with the State-Trait Anxiety Inventory (STAI-6), societal participation was assessed with the Utrecht Scale for Evaluation of Rehabilitation-Participation (USER-P), and HRQoL was assessed using the Short Form 36 (SF-36). Clinical and demographic data were retrieved from medical files. Sex was defined as sex assigned at birth.

### Statistical Analyses

Data are presented as mean (± SD), median [interquartile range], or count (valid %), depending on data distribution, which was visually assessed by means of histograms and QsexQ-plots. Ridit scores of symptom occurrence/distress are visualized in a scatterplot. Furthermore, we compared ridit scores of symptom occurrence and symptom distress across different groups: males versus females, recipients younger than 65 years old versus those 65 years and older, and recipients less than 2 years posttransplantation versus those 2 years or more posttransplantation.

To identify potential demographic, clinical, and pharmacotherapeutic factors associated with symptom burden, linear regression analyses with symptom burden as dependent variable were performed, adjusted for age and sex, because age and sex may influence symptom perception.^7^^,^^10^^,^^11^ Potential associations of symptom burden with medication nonadherence, symptoms of depression, symptoms of anxiety, and restriction in societal participation were assessed with logistic regression analyses; potential associations of symptom burden with the frequency and satisfaction scales of societal participation and HRQoL were assessed with linear regression analyses. After crude analyses, we adjusted for the potential confounders age, sex, log_2_ time since transplantation, polypharmacy (defined as the use of more than 4 drugs), diabetes, anemia, hemoglobin, estimated glomerular filtration rate (eGFR), albumin and log_2_ N-terminal pro–B-type natriuretic peptide (NT-proBNP) levels, use of tacrolimus, cyclosporine, predniso(lo)ne, and proton pump inhibitors (PPIs). We adjusted for NT-proBNP in our analyses to account for the potential confounding effect of heart failure on the relationship between symptom burden and societal participation outcomes. Furthermore, sensitivity analyses were performed in which the burden score was calculated without the MTSOSD-59R questions related to symptoms of depression and anxiety. Potential presence of effect modification by age and sex was assessed by adding interaction terms to the linear regression models.

Results of linear regression analyses are presented in standardized β values, which are the number of standard deviations the dependent variable changes when an independent variable increases by 1 SD for continuous data or with 1 unit for dichotomous data. This standardized approach allows for a consistent comparison of the strengths of different determinants and associations. Assumptions for the regression analyses were visually checked. To meet assumptions for regression, skewed data were transformed using a binary logarithm (log_2_). However, symptom burden was square root transformed, as log transformation did not sufficiently normalize its distribution, while the square root transformation provided a better model fit. Variables with more than 5% missing data are mentioned in the footnote of Table 1. Listwise exclusion was applied in regression analyses. All analyses were performed with IBM SPSS software (version 23.0, SPSS Inc., Chicago, IL, USA), and R (version 3.5.1, Vienna, Austria). A 2-sided *P* value < 0.05 was considered statistically significant. The outcome analyses were adjusted for multiple comparisons using the Bonferroni method, based on 8 predefined hypotheses linking symptom burden to (1) medication nonadherence, (2) depressive symptoms, (3) anxiety symptoms, (4) frequency, (5) restriction, and (6) satisfaction of societal participation, (7) physical HRQoL, and (8) mental HRQoL. This resulted in an adjusted significance threshold of 0.00625 for these analyses.

**Table 1:** Characteristics of 936 Kidney Transplant Recipients

Variables	Kidney Transplant Recipients (n = 936)
Mean symptom burden	78.0 [39.3-142.8]
**Recipient**	
Female sex, n (%)	363 (38.8%)
Age at visit (years)	55.6 ± 13.0
aAlcohol consumption, n (%)	
None	342 (38.7%)
<7 units/week	357 (40.4%)
≥7 units/week	185 (20.9%)
bSmoking, n (%)	108 (12.5%)
Polypharmacy (>4 drugs), n (%)	894 (95.5%)
cDiabetes, n (%)	253 (28.6%)
Anemia, n (%)	278 (29.7%)
Iron deficiency, n (%)	214 (26.1%)
dPG-SGA, n (%)	
Stadium A	646 (91.2%)
Stadium B	51 (7.2%)
Stadium C	11 (1.6%)
Body mass index (kg/m^2^)	26.7 [23.9-30.0]
Time since transplantation (years)	2.0 [1.0-9.0]
Living donor, n (%)	536 (57.3%)
Pre-emptively transplanted, n (%)	324 (34.7%)
**Laboratory measurements**	
eGFR creatinine, mL/min/1.73 m^2^	54.8 ± 18.9
Hemoglobin, mmol/L	13.5 ± 1.8
Hs-CRP, mg/L	1.8 [0.7-4.6]
NT-proBNP, ng/L	201.0 [90.5-509.0]
Albumin, g/L	43.5 ± 3.0
**Medication**	
Calcineurin inhibitor, n (%)	
No use	160 (17.1%)
Tacrolimus	647 (69.1%)
Cyclosporine	129 (13.8%)
Extended-release tacrolimus, n (%)	208 (32.1%)
mTOR inhibitor, n (%)	
No use	903 (96.5%)
Everolimus	32 (3.4%)
Sirolimus	1 (0.1%)
Proliferation inhibitor, n (%)	
No use	124 (13.2%)
Azathioprine	87 (9.3%)
Mycophenolic acid	725 (77.5%)
Prednisone or prednisolone, n (%)	911 (97.3%)
Beta blockers, n (%)	513 (54.8%)
Proton pump inhibitor, n (%)	643 (68.7%)
Statin, n (%)	528 (65.4%)

*Note*: Normally distributed variables are presented as mean ± SD, skewed data as median [interquartile range], and categorical data as number (valid percentage).

Abbreviations: PG-SGA, patient-generated global assessment scale; eGFR, estimated glomerular filtration rate as calculated using the CKD-EPI formula; hs-CRP, high-sensitive C-reactive protein; NT-proBNP, N-terminal pro-B-type natriuretic peptide.

a Data were available in 884 patients.

b Data were available in 864 patients.

c Data were available in 884 patients.

d Data were available in 708 patients.

## Results

### Baseline Characteristics

In total, 936 KTR (38.8% female; mean ± SD age, 55.6 ± 13.0 years) were included at a median [interquartile range (IQR)] of 2.0 [1.0-9.0] years after transplantation. Patients were excluded because they completed <95% of the MTSOSD-59R (n = 33, 3.4%) or if they did not use immunosuppressive medication (n = 1, 0.1%), as shown in the flow diagram (Fig S1). Mean eGFR was 54.8±18.9 mL/min/1.73 m^2^. Detailed baseline characteristics are presented in Table 1.

### Symptom Occurrence and Symptom Distress

On average, patients reported an occurrence of 19.7 ± 10.0 symptoms. Based on the ridit scores, most occurring symptoms were tiredness (23.1% sometimes, 40.5% often, 8.7% almost always, and 5.7% always), bruises (30.7% sometimes, 21.8% often, 12.8% almost always, and 16.6% always), and lack of energy (29.2% sometimes, 40.6% often, 17.6% almost always, and 8.0% always).

**Figure 1:**
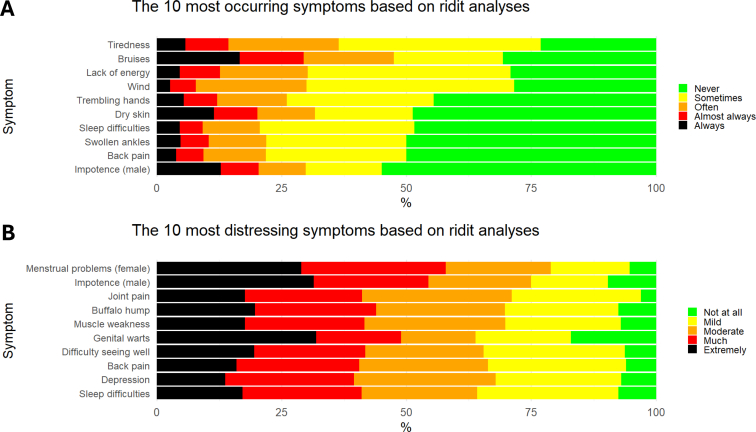
The 10 most (A) occurring and (B) distressing symptoms based on ridit scores in 936 kidney transplant recipients.

**Figure 2:**
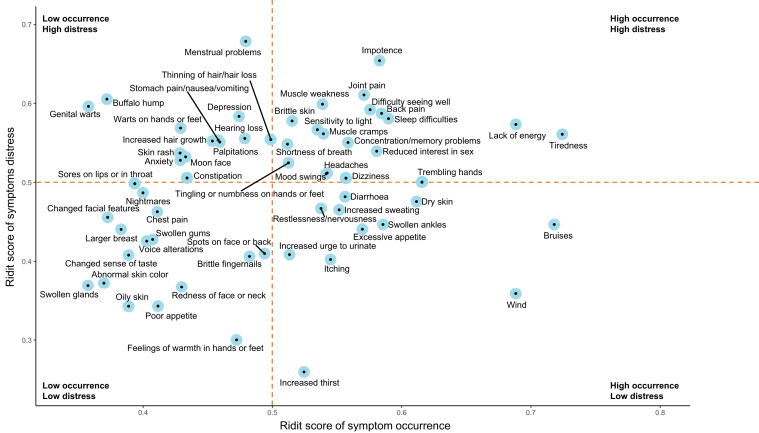
Overview of the ridit scores of symptom occurrence and symptom distress in 936 kidney transplant recipients. A ridit (relative to an identified distribution integral transformation) score ranges between 0 and 1, in which a score >0.5 indicates a higher occurrence or distress of the symptom compared with the chosen reference group, whereas a ridit score <0.5 indicates a lower occurrence or distress of the symptom compared with the chosen reference group. The reference group in this figure is the symptom occurrence or symptom distress of all symptoms of all KTR.

When present, the most distressful symptoms were menstrual problems (female; 15.8% mild, 21.1% moderate, 28.9% much, 28.9% extreme), impotence (male; 15.3% mild, 20.6% moderate, 23.0% much, 31.5% extreme), and joint pain (25.8% mild, 30.0% moderate, 23.5% much, 17.6% extreme). The ten most occurring and distressing symptoms based on ridit scores are presented in Figure 1. Lack of energy and tiredness were both frequently occurring and distressful, while bruises and wind were frequently occurring, but less distressful. In contrast, genital warts and buffalo hump were experienced as very distressful but occurred less often. A detailed overview of the ridit scores of occurrence and distress of all symptoms is presented in Figure 2.

### Symptom Perception Stratified by Sex, Age, and Time Since Transplantation

The most occurring and distressing symptoms among KTR stratified by sex, age (<65 years old vs ≥65 years old), and time since transplantation (<2 vs ≥2 years) are presented in Table 2, in which differences between groups are marked in bold.

**Table 2:** Ridit Scores of Most Occurring and Distressing Symptoms Stratified by Age, Sex, and Time After Transplantation

	Most Occurring Symptoms Stratified by Sex					Most Distressing Symptoms Stratified by Sex				
	Male (n = 573)		Female (n = 363)			Male (n = 573)		Female (n = 363)		
1	Tiredness	[0.708]	Bruises	[0.791]	1	Impotence	[0.654]	**Buffalo hump**	**[0.723]**	
2	Wind	[0.685]	Tiredness	[0.750]	2	Joint pain	[0.582]	**Increased hair growth**	**[0.691]**	
3	Bruises	[0.672]	**Dry skin**	**[0.721]**	3	Muscle weakness	[0.576]	**Genital warts**	**[0.690]**	
4	Lack of energy	[0.670]	Lack of energy	[0.717]	4	**Hearing loss**	**[0.566]**	Menstrual problems	[0.679]	
5	Trembling hands	[0.612]	Wind	[0.694]	5	**Depression**	**[0.565]**	Joint pain	[0.653]	
6	**Swollen ankles**	**[0.587]**	**Sleep difficulties**	**[0.655]**	6	Back pain	[0.556]	**Thinning of hair or hair loss**	**[0.647]**	
7	Impotence	[0.583]	**Reduced interest in sex**	[0.654]	7	Difficulty seeing well	[0.554]	Difficulty seeing well	[0.646]	
8	Back pain	[0.565]	Trembling hands	[0.622]	8	**Sensitivity to light**	**[0.549]**	Back pain	[0.629]	
9	**Difficulty seeing well**	**[0.562]**	Back pain	[0.615]	9	**Reduced interest in sex**	**[0.548]**	Muscle weakness	[0.627]	
10	**Joint pain**	**[0.556]**	**Increased sweating**	**[0.600]**	10	**Sleep difficulties**	[0.546]	**Lack of energy**	**[0.624]**	

	Most Occurring Symptoms Stratified by Age					Most Distressing Symptoms Stratified by Age			
	<65 years old (n = 669)		≥65 years old (n = 267)			<65 years old (n = 669)		≥65 years old (n = 267)	
1	Tiredness	[0.733]	Bruises	[0.793]	1	Impotence (male)	[0.686]	Impotence (male)	[0.609]
2	Wind	[0.707]	Tiredness	[0.703]	2	Menstrual problems (female)	[0.679]	Joint pain	[0.569]
3	Lack of energy	[0.693]	**Impotence (male)**	**[0.686]**	3	**Genital warts**	**[0.657]**	Back pain	[0.557]
4	Bruises	[0.688]	Lack of energy	[0.676]	4	Muscle weakness	[0.634]	Buffalo hump	[0.553]
5	**Trembling hands**	**[0.639]**	Wind	[0.642]	5	Joint pain	[0.625]	**Brittle skin**	**[0.552]**
6	**Excessive appetite**	**[0.604]**	Dry skin	[0.638]	6	Buffalo hump	[0.615]	Difficulty seeing well	[0.550]
7	Dry skin	[0.601]	**Reduced interest in sex**	**[0.612]**	7	Difficulty seeing well	[0.607]	**Increased hair growth**	**[0.547]**
8	Sleep difficulties	[0.598]	**Swollen ankles**	**[0.604]**	8	**Depression**	[0.**605]**	**Sleep difficulties**	**[0.531]**
9	**Difficulty seeing well**	**[0.583]**	Back pain	[0.591]	9	Back pain	[0.599]	**Warts on hands or feet**	**[0.527]**
10	Back pain	[0.582]	Sleep difficulties	[0.569]	10	**Lack of energy**	**[0.599]**	Muscle weakness	[0.512]

	Most Occurring Symptoms Stratified by Time After Transplantation					Most Distressing Symptoms Stratified by Time After Transplantation			
	<2 years after transplantation (n = 313)		≥2 years after transplantation (n = 623)			<2 years after transplantation (n = 313)		≥2 years after transplantation (n = 623)	
1	Tiredness	[0.688]	Bruises	[0.752]	1	Menstrual problems (female)	[0.714]	Impotence (male)	[0.672]
2	Trembling hands	[0.668]	Tiredness	[0.743]	2	Impotence (male)	[0.623]	Menstrual problems (female)	[0.664]
3	Wind	[0.660]	Lack of energy	[0.707]	3	**Thinning of hair or hair loss**	**[0.600]**	**Buffalo hump**	**[0.622]**
4	Lack of energy	[0.651]	Wind	[0.702]	4	**Depression**	**[0.599]**	Joint pain	[0.618]
5	Bruises	[0.650]	Dry skin	[0.627]	5	Joint pain	[0.594]	Muscle weakness	[0.606]
6	**Excessive appetite**	**[0.621]**	**Back pain**	**[0.601]**	6	Genital warts	[0.591]	Genital warts	[0.598]
7	**Impotence (male)**	**[0.606]**	**Swollen ankles**	**[0.594]**	7	Difficulty seeing well	[0.589]	**Brittle skin**	**[0.597]**
8	**Sleep difficulties**	**[0.601]**	Trembling hands	[0.590]	8	**Sleep difficulties**	**[0.589]**	**Back pain**	**[0.596]**
9	Dry skin	[0.581]	**Reduced interest in sex**	**[0.589]**	9	Muscle weakness	[0.584]	Difficulty seeing well	[0.593]
10	**Concentration or memory problems**	**[0.579]**	**Joint pain**	**[0.588]**	10	**concentration or memory problems**	**[0.581]**	**Warts on hands or feet**	**[0.577]**

*Note*: A ridit (relative to an identified distribution integral transformation) score ranges between 0 and 1, in which a score >0.5 indicates a higher occurrence or distress of the symptom compared with the chosen reference group, whereas a ridit score <0.5 indicates a lower occurrence or distress of the symptom compared with the chosen reference group. The reference groups in this table represent the overall occurrence or distress scores of all symptoms within each specific subgroup. For example, in the case of males, the reference group consists of the occurrence or distress scores of all symptoms reported by male participants. Bold symptoms are specific for that subgroup.

Abbreviations: KTR, kidney transplant recipients.

Compared with females, males reported high occurrence of swollen ankles, difficulty seeing well, and joint pain as well as high distress of hearing loss, depression, sensitivity to light, reduced interest in sex, and sleep difficulties. Compared with males, females reported high occurrence of dry skin, sleep difficulties, reduced interest in sex, and increased sweating as well as high distress of buffalo hump, increased hair growth, genital warts, thinning of hair/hair loss, and lack of energy.

Compared with older KTR, younger KTR reported high occurrence of trembling hands, excessive appetite and difficulty seeing well as well as high distress of genital warts, depression and lack of energy. Compared with younger KTR, older KTR reported high occurrence of impotence, reduced interest in sex, and swollen ankles as well as high distress of brittle skin, increased hair growth, sleep difficulties, and warts on hands or feet.

Compared with KTR ≥2 years after transplantation, KTR <2 years after transplantation reported high occurrence of excessive appetite, impotence, sleep difficulties, and concentration/memory problems as well as high distress of thinning of hair/hair loss, depression, sleep difficulties and concentration/memory problems. Compared with KTR <2 years after transplantation, KTR ≥2 years after transplantation reported high occurrence of back pain, swollen ankles, reduced interest in sex, and joint pain as well as high distress of buffalo hump, brittle skin, back pain, and warts on hands or feet.

### Factors Associated With Symptom Burden

Median symptom burden was 78.0 [39.3-142.8]. Four (0.4%) patients reported zero symptom burden. In linear regression analyses adjusted for age, being female (*P* < 0.001) was associated with higher symptom burden (Table 3). In further analyses adjusted for both age and sex, a worse nutritional status (*P* < 0.001 for PG-SGA stadium B; *P* = 0.002 for PG-SGA stadium C), anemia (*P* = 0.01), and longer time since transplantation (*P* = 0.01) were associated with higher symptom burden. In contrast, higher eGFR, higher hemoglobin concentration, and higher albumin concentration were associated with lower symptom burden (*P* = 0.04, *P* < 0.001, *P* = 0.006, respectively). Lastly, cyclosporine use and PPI use were associated with higher symptom burden (*P* = 0.005 and *P* < 0.001, respectively), whereas use of mycophenolic acid was associated with lower symptom burden (*P* = 0.002).

**Table 3:** Linear regression analyses with square root symptom burden as dependent variable, adjusted for sex and age, in 936 kidney transplant recipients

Variables	bSquare root symptom burden, adjusted for sex and age	
	St.β (95% CI)	*P* Value
**Recipient**		
Female sex^a^	**0.49 (0.36 to 0.62)**	**<0.001**
Age at visit^a^	–0.04 (–0.11 to 0.02)	0.16
Alcohol consumption		
None	Ref.	*-*
<7 units/week	0.06 (–0.08 to 0.21)	0.40
≥7 units/week	–0.07 (–0.25 to 0.11)	0.44
Smoking	–0.03 (–0.23 to 0.16)	0.75
Polypharmacy (>4 drugs)	0.20 (–0.11 to 0.50)	0.21
Diabetes	0.07 (–0.07 to 0.22)	0.33
Anemia	**0.17 (0.03 to 0.31)**	**0.01**
Iron deficiency	0.12 (–0.03 to 0.27)	0.11
PG-SGA		
Stadium A	*Ref.*	-
Stadium B	**0.83 (0.56 to 1.10)**	**<0.001**
Stadium C	**0.90 (0.33 to 1.46)**	**0.002**
Body mass index (kg/m^2^)^b^	0.03 (-0.03 to 0.10)	0.30
Time since transplantation (years)^b^	**0.08 (0.02 to 0.14)**	**0.01**
Living donor	–0.07 (–0.20 to 0.06)	0.29
Pre-emptive	0.05 (–0.08 to 0.18)	0.46
**Laboratory measurements**		
eGFR Creatinine (mL/min/1.73 m^2^)	**–0.07 (–0.13 to –0.00)**	**0.04**
Hemoglobin (mmol/L)	**–0.13 (–0.19 to –0.06)**	**<0.001**
Hs-CRP (mg/L)^b^	0.01 (–0.06 to 0.07)	0.84
NT-proBNP (ng/L)^b^	**0.09 (0.02 to 0.15)**	**0.01**
Albumin (g/L)	**–0.09 (–0.16 to –0.03)**	**0.006**
**Medication**		
Calcineurin inhibitor		
No use	*Ref.*	-
Tacrolimus	–0.08 (–0.25 to 0.09)	0.35
Cyclosporine	**0.32 (0.10 to 0.55)**	**0.005**
Extended-release tacrolimus	0.02 (–0.14 to 0.18)	0.77
MTOR inhibitor		
No use	*Ref.*	-
Everolimus	0.02 (–0.33 to 0.36)	0.92
Sirolimus	0.34 (–1.56 to 2.25)	0.72
Proliferation inhibitor		
No use	*Ref.*	-
Azathioprine	–0.15 (–0.41 to 0.12)	0.28
Mycophenolic acid	**–0.30 (–0.48 to –0.11)**	**0.002**
Prednisone or prednisolone	0.19 (–0.19 to 0.58)	0.32
Beta blockers	0.04 (–0.09 to 0.17)	0.54
Proton pump inhibitor	**0.25 (0.12 to 0.39)**	**<0.001**
Statin	–0.09 (–0.22 to 0.04)	0.16

*Note*: Bold type indicates significance of results.

Abbreviations: 95% CI, 95% confidence interval; PG-SGA, patient-generated global assessment scale; eGFR, estimated glomerular filtration rate as calculated using the CKD-EPI formula; hs-CRP, high-sensitive C-reactive protein; NT-proBNP, N-terminal pro-B-type natriuretic peptide.

a Only adjusted for the other variables.

b Outcome variable was log_2_-transformed. Symptom burden was square root transformed to fulfill the assumptions of the linear regression analyses. Square root symptom burden values were standardized to compare strengths of different determinants and associations. A positive value indicates higher symptom burden whereas a lower value indicates lower symptom burden.

### Medication Nonadherence, Symptoms of Depression, Symptoms of Anxiety, and Societal Participation

In total, 605 (38.1%) patients reported medication nonadherence. Moreover, 129 (8.0%) reported moderate to severe symptoms of depression, and 238 (26.0%) symptoms of anxiety. In logistic regression analyses, higher symptom burden was associated with more medication nonadherence (OR per SD increase 1.27, 95% CI 1.12-1.46, *P* < 0.001), more symptoms of depression (OR per SD increase 4.25, 95% CI, 3.13-5.77, *P* < 0.001), and more symptoms of anxiety (OR per SD increase 2.61, 95% CI, 2.19-3.11, *P* < 0.001). Sensitivity analyses in which the burden score was calculated without the MTSOSD-59R questions related to symptoms of depression/anxiety, showed similar results (Table S3). Mean frequency and satisfaction scores of societal participation were 32.4 ± 10.6 and 77.4 ± 16.6, respectively, and a restriction in societal participation was reported by 498 (58.2%) KTR. In linear and logistic regression analyses, higher symptom burden was associated with a lower frequency of activities (st.β -0.13, 95% CI –0.20 to –0.06, *P* < 0.001), more restrictions in societal participation (OR per SD 0.28, 95% CI 0.23-0.34, *P* < 0.001), and less satisfaction (st.β -0.33, 95% CI –0.39 to –0.27, *P* < 0.001). All results remained after adjustment for age, sex, log_2_ time since transplantation, polypharmacy, diabetes, anemia, hemoglobin level, eGFR, albumin level, log_2_ NT-proBNP level, tacrolimus use, cyclosporine use, predniso(lo)ne use, and PPI use (model 1, Table 4).

**Table 4:** Logistic and linear regression analyses of square root symptom burden with medication nonadherence, symptoms of depression, symptoms of anxiety, participation (activity, restriction and satisfaction), and physical and mental HRQoL as dependent variables in 936 kidney transplant recipients

	aMedication Nonadherence (n = 912)		aSymptoms of Depression (n = 908)		aSymptoms of Anxiety (n = 917)	
Model	OR per SD (95% CI)	*P* Value	OR per SD (95% CI)	*P* Value	OR per SD (95% CI)	*P* Value
Crude	1.27 (1.12 to 1.46)	<0.001	4.25 (3.13 to 5.77)	<0.001	2.61 (2.19 to 3.11)	<0.001
Model 1	1.38 (1.18 to 1.60)	<0.001	4.98 (3.46 to 7.17)	<0.001	2.80 (2.30 to 3.40)	<0.001

	bFrequency of Activities (n = 860)		aRestrictions (n = 855)		bSatisfaction (n = 856)	
Model	St.β (95% CI)	*P* Value	OR per SD (95% CI)	*P* Value	St.β (95% CI)	*P* Value
Crude	–0.13 (–0.20 to –0.06)	<0.001	0.28 (0.23 to 0.34)	<0.001	–0.33 (–0.39 to –0.27)	<0.001
Model 1	–0.16 (–0.23 to –0.09)	<0.001	0.25 (0.20 to 0.32)	<0.001	–0.32 (–0.39 to –0.25)	<0.001

	bPhysical Component Score (n = 740)		bMental Component Score (n = 740)	
Model	St.β (95% CI)	*P* Value	St.β (95% CI)	*P* Value
Crude	–0.55 (–0.61 to –0.49)	<0.001	–0.53 (–0.59 to –0.47)	<0.001
Model 1	–0.53 (–0.59 to –0.47)	<0.001	–0.53 (–0.60 to –0.46)	<0.001

*Notes*: Model 1 was adjusted for age, sex, log_2_ time since transplantation, polypharmacy, diabetes, anemia, hemoglobin, eGFR, albumin, log_2_ NT-proBNP, tacrolimus, cyclosporine, predniso(lo)ne and proton pump inhibitors.

Abbreviations: OR, odds ratio; SD, standard deviation; 95% CI, 95% confidence interval.

a Logistic regression analysis was used to assess potential associations. Symptom burden was square root transformed to fulfill the assumptions of the linear regression analyses.

b Linear regression analyses were used to assess potential associations. Symptom burden was square root transformed to fulfill the assumptions of the linear regression analyses.

### Health-related Quality of Life

The mean physical component summary (PCS) and mental (MCS) were 68.9 ± 21.9 and 76.5 ± 17.4, respectively. In linear regression analyses, higher symptom burden was associated with lower PCS (st.β -0.55, 95% CI –0.61 to –0.49, *P* < 0.001), as well as lower MCS (st.β –0.53, 95% CI –0.59 to –0.47, *P* < 0.001), which remained after adjustment (Table 4).

### Effect Modification

No interactions of age and sex were present for the associations of burden score with medication nonadherence, symptoms of depression/anxiety, societal participation, and HRQoL.

### Discussion

This large cross-sectional study provides clinicians valuable insights of symptom experience in KTR. The most occurring symptoms were tiredness, bruises, and lack of energy, whereas the most distressful symptoms were menstrual problems, impotence, and joint pain. Low nutritional status, being female, cyclosporine use, and PPI use were strongest associated with higher symptom burden, whereas mycophenolic acid use, higher hemoglobin levels, higher albumin levels, and higher eGFR were associated with lower symptom burden. Additionally, higher symptom burden was associated with medication nonadherence, symptoms of depression/anxiety, poorer societal participation, and lower HRQoL.

Previous research demonstrated that different study populations experience different symptoms as most occurring and distressing.^7^ In accordance with a recent study in another Dutch population of 631 KTR, our KTR population experienced tiredness and bruises as most occurring and the sex-specific symptoms impotence and menstrual problems as most distressing.^8^ Tiredness is a common and burdensome symptom in patients with end-stage kidney disease, potentially impairing daily functioning, emotional well-being, and HRQoL.^23^ Our results show that this symptom remains important even after successful kidney transplantation. KTR studies from Switzerland and Korea showed similar results with tiredness being the most prevalent experienced symptom.^11^^,^^12^ However, joint pain and impotence gave the most distress to the Swiss KTR, whereas lack of energy gave the most distress to the Korean KTR.^11^^,^^12^ Possible explanations for these differences in symptom experience may be differences in cultural values, health care access and environmental factors.^6^ Factors such as sex, age, and time since transplantation may influence this symptom experience. Another Dutch study already compared symptom occurrence between groups, and showed similar results.^8^ We noticed that male KTR reported high distress from psychological symptoms (ie, depression, reduced interest in sex), compared with female KTR who reported high distress from aesthetic symptoms such as thinning of hair/hair loss and increased hair growth. Younger KTR also reported high distress of psychological symptoms (ie, depression, lack of energy), whereas older KTR various other types of symptoms as highly distressing. KTR <2 years after transplantation reported high distress from aesthetic and psychological symptoms, whereas KTR ≥2 years after transplantation also reported various other symptoms as highly distressing (ie, buffalo hump, brittle skin, back pain, and warts on hands or feet). A possible reason for this may be that the ≥2 years after transplantation group contained significantly older KTR or that longer exposition to steroids that may cause high distress of buffalo hump in the ≥2 years after transplantation group.

Combining symptom occurrence and distress into symptom burden facilitated extensive exploration of potential determinants. We confirm findings of studies showing that women more frequently experience symptoms, and report more symptom distress.^7^^,^^10^^,^^11^ Moons et al^10^ found that younger patients had higher symptom distress; however, we observed no association between age and symptom burden. We observed associations of anemia and a decreased kidney function with higher symptom burden. In addition, a worse nutritional status was associated with higher symptom burden, in accordance with a study that associated higher protein intake with higher HRQoL in KTR.^24^ Furthermore, higher NT-proBNP levels and longer time since transplantation were associated with higher symptom burden, which aligns with a study in heart transplant recipients who perceived more symptom distress with increasing time since transplantation.^25^ Furthermore, we found that cyclosporine use was associated with higher symptom burden, suggesting a more favorable side effect profile for tacrolimus.^26^ Interestingly, extended-release tacrolimus was not associated with symptom burden, despite the hypothesis that extended-release tacrolimus may result in fewer symptoms because of lower peak concentrations.^27^ Mycophenolic acid was associated with lower symptom burden, possibly because patients who cannot tolerate it are switched to azathioprine.^28^ An alternative explanation is that mycophenolic acid is more selective than azathioprine. Lastly, PPI use was associated with higher symptom burden, which is in accordance with a previous study that shows associations of PPI use with fatigue and lower HRQoL among KTR.^29^

Our results confirm the associations of higher symptom burden with more medication nonadherence, symptoms of depression, and lower HRQoL.^10^^,^^12^, ^13^, ^14^, ^15^ Furthermore, we established associations of higher symptom burden with more symptoms of anxiety and lower societal participation. These patient-reported outcomes may be entangled and influence each other, and together they are important predictors of long term outcomes after transplantation such as hospitalization, graft survival and mortality.^14^^,^^16^, ^17^, ^18^, ^19^ We propose routinely assessing symptom status—eg, through previsit questionnaires—to help clinicians save consultation time and identify symptoms that patients may not report spontaneously, such as sexual problems, which can significantly affect HRQoL.

It is important to know how patients perceive their symptoms, so health care providers may improve patient well-being by focusing on adequate support, and by tailoring (immunosuppressive) treatment to the recipients’ needs. Patients who perceive all their symptoms as treatable may have a higher HRQoL.^30^ In addition, providing adequate support may improve psychosocial adaptation.^31^ Therefore, interventions to improve symptom status could focus on enhancing support in addition to adequate tailoring of medication regimens.

A strength of our study is the high cohort participation rate and questionnaire response rate, resulting in a large representative study population.^32^ Moreover, the extensive availability of demographic, clinical, and pharmacotherapeutic data allowed for exploration of potential determinants of symptom burden. However, longitudinal analyses are needed to draw conclusions regarding causality of our findings. Inherently, we do not know whether the symptoms are side effects of medication or whether they are caused by underlying pathology. In addition, because women tend to report a higher symptom burden, and we used ridit analyses with all KTR as reference group, there might be an overestimation of men’s symptom burden and an underestimation of women’s symptom burden. Lastly, the single center study design causes limited generalizability, while symptom experience can vary per region because of differences in environmental factors, access to health care, and cultural determinants.

In conclusion, we identified the most occurring symptoms and the most distressing symptoms among stable KTR. The strongest associations with higher symptom burden were female sex, malnutrition, cyclosporine use and PPI use. Higher symptom burden was associated with medication nonadherence, symptoms of depression, symptoms of anxiety, poorer societal participation, and lower HRQoL. Future longitudinal studies may focus on the most occurring and distressing symptoms.

## Supplemental Materials

Supplementary File (PDF) Figures S1-S2; Tables S1-S3; Item S1.
