# Supplementary material for: Symptom Burden and Patient-Reported Outcomes in Kidney Transplant Recipients: Results From the TransplantLines Biobank and Cohort Studies
Source: Kidney Med. 2025 Oct 31;8(1):101168. doi: 10.1016/j.xkme.2025.101168 (PMC12771093; doi:10.1016/j.xkme.2025.101168)
Supplement: Supplementary File (PDF) — Figures S1-S2; Tables S1-S3; Item S1. [file mmc1.pdf]

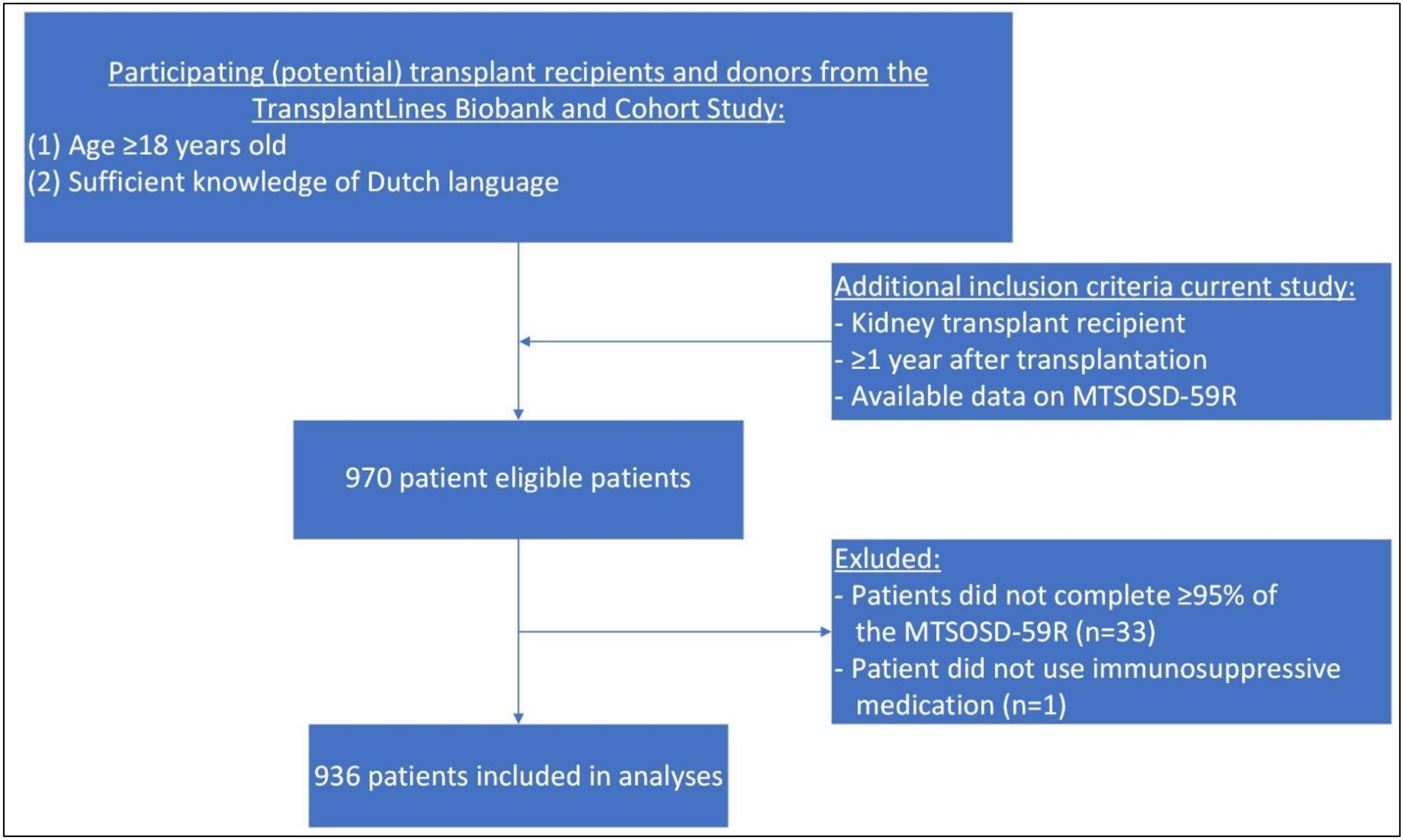

Figure S1 | Flow diagram of patient inclusion. Abbreviations: MTSOSD-59R: the revised 59-item Modified Transplant Symptom Occurrence and Symptom Distress Scale

Table S1 | Overview of the revised 59-item Modified Transplant Symptom Occurrence and Symptom Distress Scale items

1) Itching	31) Tingling or numbness of hands or feet
2) Chest pain	32) Back pain
3) Wind	33) Brittle skin
4) Increased thirst	34) Anxiety
5) Restlessness\nervousness	35) Mood swings
6) Hearing loss	36) Headaches
7) Abnormal skin color	37) Changed facial features
8) Increased sweating	38) Buffalo hump
9) Redness of face or neck	39) Concentration or memory problems
10) Brittle fingernails	40) Warts on hands or feet
11) Larger breasts	41) Increased hair growth
12) Sores on lips or in mouth	42) Sleep difficulties
13) Voice alterations	43) Muscle weakness
14) Oily skin	44) Changed sense of taste
15) Dizziness	45) Poor appetite
16) Trembling hands	46) Tiredness
17) Increased urge to urinate	47) Lack of energy
18) Feeling of warmth in hands or in feet	48) Stomach complaints/nausea/vomiting
19) Bruises	49) Joint pain
20) Genital warts	50) Skin rash
21) Spots on face or back	51) Muscle cramps
22) Excessive appetite	52) Nightmares
23) Depression	53) Shortness of breath
24) Swollen gums	54) Dry skin
25) Swollen glands	55) Palpitations
26) Thinning of hair/hair loss	56) Constipation
27) a. Menstrual problems (female) *OR* b. impotence (male)	57) Difficulty seeing well
28) Moon face	58) Reduced interest in sex
29) Swollen ankles	59) Sensitivity to light
30) Diarrhea	

Table S2 | Detailed description of assessment of outcome variables and additional data collection

**Outcome**	**assessment**
Medication nonadherence	Medication nonadherence was assessed using the Basel Assessment of Adherence to Immunosuppressive Medication Scale (BAASIS). This validated questionnaire contains 5 questions that assess the three phases of medication use according to the ABC taxonomy of medication nonadherence: initiation, implementation and persistence.[1] Patients were considered nonadherent if they replied ‘yes’ to at least one question.
Symptoms of depression	Depressive symptoms were assessed using the clinically validated Patient Health Questionnaire (PHQ-9).[2] The PHQ-9 evaluates the 9 DSM-IV criteria, and each criterion can be graded with a score from ‘0’ (not at all) to ‘3’ (nearly every day). Patients with a PHQ-9 score ≥10 are considered to have moderate to severe symptoms of depression.[3,4]
Symptoms of anxiety	Symptoms of anxiety were measured with the short six-item version of The State-Trait Anxiety Inventory (STAI-6).[5,6] This questionnaire is designed to measure current state of anxiety (how much worry, tension, or apprehension does the participant experience), and how much of the anxiety is reflected in the person’s personality. A score ≥40 was considered as severe symptoms of anxiety.[7]
Societal participation	Societal participation was assessed by the Utrecht Scale for Evaluation of Rehabilitation-Participation (USER-P). This validated questionnaire comprises 31 items in three scales: frequency, restrictions, and satisfaction.[8] For each scale, a sum score was calculated and converted to a score ranging from 0 to 100; higher scores indicate better levels of participation (higher frequency, less restrictions, higher satisfaction). A score <100 was interpreted as the patient having restrictions, because there was a ceiling effect (i.e. multiple patients had no restrictions).
HRQoL	HRQoL was assessed using the Short Form 36 (SF-36) health survey.[9] The SF-36 health survey evaluates several health domains from which a physical component score (PCS) and a mental component score (MCS) were derived. The PCS was calculated by averaging the physical functioning, role limitations due to physical health, pain, and general health perceptions domains, whereas the MCS was calculated by averaging role limitations due to emotional problems, vitality, emotional well-being, and social functioning domains. Higher scores indicate higher HRQoL.[10,11]
Additional data collection	Clinical and transplant-related parameters were obtained from electronic patient files. Medication usage was subsequently verified and corrected with the patient. Body weight and height were measured with participants wearing indoor clothing and no shoes. Body mass index (BMI) was calculated as weight in kilograms divided by height in square meters (kg/m^2). Smoking status and alcohol use were assessed by questionnaires and categorized into yes/no and none/<7 units per week/≥7 units per week, respectively. Laboratory measurements were performed using routine laboratory techniques (Roche, Basel, Switzerland). Diabetes was defined based on use of antidiabetic drugs, fasting glucose levels ≥7.0 mmol/L, and/or HbA1c levels ≥6.5%.[12] The

	creatinine-based CKD-EPI equation was used to calculate estimated glomerular filtration rate (eGFR).[13] According to WHO criteria, anemia was defined as a hemoglobin level <13 g/dL for men and <12 g/dL for women.[14] Iron deficiency was defined as both transferrin saturation <20% and ferritin <100 µg/L.[15]

Abbreviations: HRQoL: health-related quality of life.

Table S3 | #Sensitivity logistic and linear regression analyses of square root symptom burden with medication nonadherence, symptoms of depression, severe symptoms of anxiety, and participation (activity, restriction and satisfaction) as dependent variables in 936 kidney transplant recipients

	[1]Medication nonadherence (n=912)		[1]Symptoms of depression (n=908)		[1]Symptoms of anxiety (n=917)	
Model	OR per SD (95%CI)	P-value	OR per SD (95%CI)	P-value	OR per SD (95%CI)	P-value
Crude	1.27 (1.11 to 1.45)	<0.001	4.13 (3.06 to 5.57)	<0.001	2.60 (2.19 to 3.10)	<0.001
Model 1	1.37 (1.17 to 1.59)	<0.001	4.78 (3.34 to 6.83)	<0.001	2.77 (2.28 to 3.37)	<0.001

	[2]Frequency of activities (n=860)		[1]Restrictions (n=855)		[2]Satisfaction (n=856)	
Model	St.β (95%CI)	P-value	OR per SD (95%CI)	P-value	St.β (95%CI)	P-value
Crude	-0.13 (-0.20 to -0.06)	<0.001	0.28 (0.23 to 0.35)	<0.001	-0.33 (-0.40 to -0.27)	<0.001
Model 1	-0.16 (-0.23 to -0.09)	<0.001	0.25 (0.20 to 0.32)	<0.001	-0.32 (-0.39 to -0.25)	<0.001

Abbreviations: 95%CI: 95% confidence interval. **Model 1**: adjusted for age, sex, $\log_2$ time since transplantation, polypharmacy, diabetes, anemia, hemoglobin, eGFR, albumin, $\log_2$ NT-proBNP, tacrolimus, cyclosporine, predniso(lo)ne, and proton pump inhibitors. #Square root symptom burden was calculated without question 23 (depression), and 34 (anxiety) of the revised 59-item Modified Transplant Symptom Occurrence and Symptom Distress Scale. [1]Logistic and [2]linear regression analyses were used to assess potential associations.

Strobe checklist

STROBE Statement—Checklist of items that should be included in reports of ***cross-sectional studies***

	Item No	Recommendation	Page No
**Title and abstract**	1	(*a*) Indicate the study's design with a commonly used term in the title or the abstract	3
		(*b*) Provide in the abstract an informative and balanced summary of what was done and what was found	3-4
**Introduction**			
Background/rationale	2	Explain the scientific background and rationale for the investigation being reported	5-6
Objectives	3	State specific objectives, including any prespecified hypotheses	5-6
**Methods**			
Study design	4	Present key elements of study design early in the paper	7
Setting	5	Describe the setting, locations, and relevant dates, including periods of recruitment, exposure, follow-up, and data collection	7
Participants	6	(*a*) Give the eligibility criteria, and the sources and methods of selection of participants	7
Variables	7	Clearly define all outcomes, exposures, predictors, potential confounders, and effect modifiers. Give diagnostic criteria, if applicable	7-8
Data sources/ measurement	8*	For each variable of interest, give sources of data and details of methods of assessment (measurement). Describe comparability of assessment methods if there is more than one group	*7-8*
Bias	9	Describe any efforts to address potential sources of bias	9
Study size	10	Explain how the study size was arrived at	7
Quantitative variables	11	Explain how quantitative variables were handled in the analyses. If applicable, describe which groupings were chosen and why	9
Statistical methods	12	(*a*) Describe all statistical methods, including those used to control for confounding	9-10
		(*b*) Describe any methods used to examine subgroups and interactions	9-10
		(*c*) Explain how missing data were addressed	10
		(*d*) If applicable, describe analytical methods taking account of sampling strategy	NA
		(*e*) Describe any sensitivity analyses	9

**Results**			
Participants	13*	(a) Report numbers of individuals at each stage of study—eg numbers potentially eligible, examined for eligibility, confirmed eligible, included in the study, completing follow-up, and analysed	11
		(b) Give reasons for non-participation at each stage	11
		(c) Consider use of a flow diagram	11
Descriptive data	14*	(a) Give characteristics of study participants (eg demographic, clinical, social) and information on exposures and potential confounders	11, 23
		(b) Indicate number of participants with missing data for each variable of interest	23
Outcome data	15*	Report numbers of outcome events or summary measures	28
Main results	16	(*a*) Give unadjusted estimates and, if applicable, confounder-adjusted estimates and their precision (eg, 95% confidence interval). Make clear which confounders were adjusted for and why they were included	26-28
		(*b*) Report category boundaries when continuous variables were categorized	Supl.
		(*c*) If relevant, consider translating estimates of relative risk into absolute risk for a meaningful time period	NA
Other analyses	17	Report other analyses done—eg analyses of subgroups and interactions, and sensitivity analyses	13
**Discussion**			
Key results	18	Summarise key results with reference to study objectives	14
Limitations	19	Discuss limitations of the study, taking into account sources of potential bias or imprecision. Discuss both direction and magnitude of any potential bias	16
Interpretation	20	Give a cautious overall interpretation of results considering objectives, limitations, multiplicity of analyses, results from similar studies, and other relevant evidence	14-17
Generalisability	21	Discuss the generalisability (external validity) of the study results	16
**Other information**			
Funding	22	Give the source of funding and the role of the funders for the present study and, if applicable, for the original study on which the present article is based	18
